# Slowdown in Life Expectancy Improvements for European Countries From 2000 to 2019

**DOI:** 10.1093/geroni/igab046.572

**Published:** 2021-12-17

**Authors:** Nicholas Steel, John Ford, Jurgen Schmidt

**Affiliations:** 1 University of East Anglia, Norwich Norfolk, England, United Kingdom; 2 Cambridge University, Cambridge, England, United Kingdom; 3 Public Health England, London, England, United Kingdom

## Abstract

Life expectancy improvements have slowed across Europe since around 2010 for unknown reasons. We aimed to assess the contribution of specific conditions and risk factors to changes in life expectancy. We compared Global Burden of Disease (GBD) 2019 estimates for life expectancy at birth, years of life lost to premature mortality (YLLs) and population attributable fractions (PAFs) for risk factors, for 17 European Economic Area (EEA) countries from 2000 to 2010 and from 2010 to 2019. All 17 countries experienced a slowdown in life expectancy improvements after 2010, after decades of improvement. Denmark experienced the smallest drop in improvement from 2000 to 2010 compared to 2010 to 2019 (0.75 years drop), followed by Norway (0.79), Iceland (0.86), Finland and Sweden (both 0.89). The 5 countries with the largest drop in improvement were Spain (1.6 years drop), the Netherlands (1.88), Portugal (1.92), the United Kingdom (UK) (2.13), and Ireland (2.77). Ischaemic heart disease and stroke made the biggest contribution to the slowdown in life expectancy. Important risk factors for mortality varied by country and included tobacco, drug and alcohol use, and high fasting plasma glucose. The Nordic countries have maintained improvements in life expectancy substantially better than other European countries. The different patterns in different countries suggest multiple factors are contributing to the changes, including specific conditions, risks and behaviours, and broader societal determinants of health. Large scale, international, co-ordinated research is needed to better understand these changes and inform policy actions, particularly as the COVID-19 pandemic will increase international differences.

